# Capacity-Limited Failure in Approximate Nearest Neighbor Search on Image Embedding Spaces

**DOI:** 10.3390/jimaging12020055

**Published:** 2026-01-25

**Authors:** Morgan Roy Cooper, Mike Busch

**Affiliations:** Anderson College of Business and Computing, Regis University, Denver, CO 80221, USA; mbusch@regis.edu

**Keywords:** capacity-limited failure, image embeddings, approximate nearest neighbors, *k*-NN, HNSW, neighborhood geometry, similarity search, intrinsic dimensionality, barycenter shift, neighborhood overlap

## Abstract

Similarity search on image embeddings is a common practice for image retrieval in machine learning and pattern recognition systems. Approximate nearest neighbor (ANN) methods enable scalable similarity search on large datasets, often approaching sub-linear complexity. Yet, little empirical work has examined how ANN neighborhood geometry differs from that of exact *k*-nearest neighbors (*k*-NN) search as the neighborhood size increases under constrained search effort. This study quantifies how approximate neighborhood structure changes relative to exact *k*-NN search as *k* increases across three experimental conditions. Using multiple random subsets of 10,000 images drawn from the STL-10 dataset, we compute ResNet-50 image embeddings, perform an exact *k*-NN search, and compare it to a Hierarchical Navigable Small World (HNSW)-based ANN search under controlled hyperparameter regimes. We evaluated the fidelity of neighborhood structure using neighborhood overlap, average neighbor distance, normalized barycenter shift, and local intrinsic dimensionality (LID). Results show that exact *k*-NN and ANN search behave nearly identically when efSearch>k. However, as the neighborhood size grows and efSearch remains fixed, ANN search fails abruptly, exhibiting extreme divergence in neighbor distances at approximately k≈2–3.5×efSearch. Increasing index construction quality delays this failure, and scaling search effort proportionally with neighborhood size (efSearch=α×k with α≥1) preserves neighborhood geometry across all evaluated metrics, including LID. The findings indicate that ANN search preserves neighborhood geometry within its operational capacity but abruptly fails when this capacity is exceeded. Documenting this behavior is relevant for scientific applications that approximate embedding spaces and provides practical guidance on when ANN search is interchangeable with exact *k*-NN and when geometric differences become nontrivial.

## 1. Introduction

Similarity search refers to the task of retrieving items from a dataset that are most similar to a given query. These elements are typically represented as vector embeddings and compress complex data, such as images, documents, or audio, into points on an embedding manifold. Similarity between points is determined by the geometric distance in an embedding space, and neighborhoods are usually defined as the set of *k* nearest points to a given search query, or equivalently, as all items within a fixed distance parameter.

An exact similarity search performs exhaustive pairwise comparisons between all other points in the dataset and therefore scales quadratically, making it impractical for large datasets. Indyk and Motwani [1] introduced the approximate nearest neighbor (ANN) framework to address this limitation, which sacrifices a small amount of accuracy to achieve substantial reductions in complexity, producing algorithms that often approach sub-linear complexity. ANN methods such as locality-sensitive hashing (LSH) [1] and Hierarchical Navigable Small World (HNSW) [2] have become the industry standard for large-scale vector retrieval. Although recall for ANN search has been well documented [3,4], relatively little empirical evidence is available on how ANN hyperparameters affect geometric neighborhood structure, particularly as neighborhood size varies.

Filling this gap is pertinent to understanding the relationship between high-dimensional geometry, local neighborhood structure, and the approximation strategies used in modern indexing structures. Early work by Beyer et al. [5] revealed that as the dimension of embeddings grows, the distance between the nearest and furthest neighbors collapses, making it increasingly difficult to distinguish true neighborhoods. Aggarwal et al. [6] further investigated the phenomenon by analyzing Minkowski distance metrics and showed that while some Minkowski norms exhibit a higher relative contrast between nearest and farthest neighbors, the collapse of distances in high-dimensional spaces is a fundamental property of high-dimensional geometry. François et al. [7] attributed this curse of high dimensionality to the intrinsic behavior of geometry and argued that only reducing the effective dimensionality of the data itself could yield an improvement in relative contrast.

Intrinsic dimensionality [8] plays a critical role in understanding neighborhood behavior by characterizing geometric degrees of freedom in an embedding space. Amsaleg et al. [9] introduced the concept of local intrinsic dimensionality (LID) as a local measure of how rapidly the number of neighbors grows as the distance from a query point increases. Later work by Ansuini et al. [10] and Gong et al. [11] showed that deep neural networks (DNN) can compress high-dimensional data into effectively lower-dimensional structures in both the activation space and in the parameter landscape, making them powerful embedding mechanisms. Beyond the embedding models themselves, graph construction is vital for geometric approximation. Maier et al. [12] showed that different graph constructs can lead to systematically different neighborhood structures. In contrast, Ertöz et al. [13] proposed the use of a shared nearest neighbor (SNN) algorithm to stabilize clustering in varying densities. Finally, Musgrave et al. [14] highlighted several flaws in the current literature on metric learning, emphasizing the importance of a rigorous, standardized evaluation of experiments on embedding spaces.

Together, these studies reveal that neighborhood structure stability depends on more than the selected distance metric. It depends on embedding geometry, effective dimensionality, and the mechanisms and operating parameters used for approximation. Despite the widespread adoption of ANN methods, almost no empirical work has examined how approximate search deforms neighborhood structure relative to exact search as neighborhood size increases, under either a fixed or varying exploration factor.

This study fills that gap by quantifying how similarity relationships deconstruct under approximation. Using multiple random subsets of 10,000 images drawn from the STL-10 dataset [15], we compute ResNet-50 [16] image embeddings, perform an exact *k*-NN search, and perform an approximate search with an HNSW-based ANN index under controlled hyperparameter settings. We then evaluate changes in neighborhood structure using neighborhood overlap, average neighbor distance, normalized barycenter shift, and local intrinsic dimensionality (LID). The purpose of this study is not to measure recall or speed, but to gain insight into how neighborhood geometry depends on neighborhood size and search effort in approximate nearest neighbor search.

## 2. Materials and Methods

We designed this study to measure divergence in the structure of image embedding neighborhoods between exact similarity search and approximate nearest neighbor (ANN) search in a controlled environment. To quantify this, we performed exact and approximate similarity searches on the same set of image embeddings. We measured how their neighborhoods differ over a range of *k* values under controlled hyperparameter settings. The experimental process consists of five main steps: collecting a sample of images, generating image embeddings, performing an exact search, performing ANN searches under controlled parameter settings, and finally measuring the structure of the resulting neighborhoods. All searches use the same image dataset, embedding model, distance metric, and comparison metrics, while search effort parameters are varied in a controlled manner. This study does not compare different algorithms or similarity metrics, nor does it propose a new approximation methodology; instead, it focuses on quantifying how neighborhood characteristics depend on the relationship between neighborhood size and search effort.

### 2.1. Dataset

**Figure 1:**
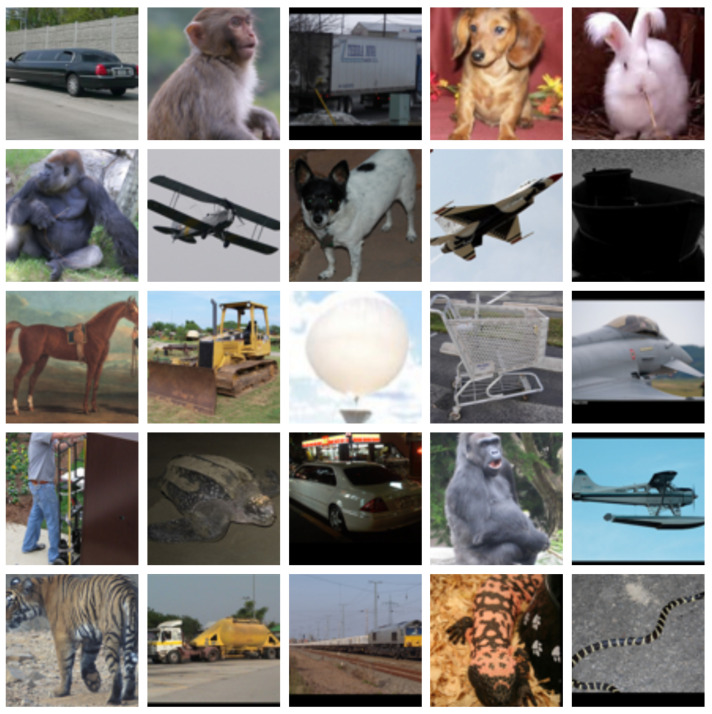
Example images from the STL-10 dataset used for embedding and nearest neighbor analysis.

We use multiple subsets of 10,000 images (Figure 1) randomly sampled without replacement from the STL-10 dataset, with the sample size chosen based on computational feasibility. STL-10 is an image recognition benchmark inspired by the CIFAR-10 dataset [17] and is considered more challenging due to its higher-resolution images (96 × 96) and greater visual diversity. The STL-10 dataset contains 10 labeled image classes—airplane, bird, car, cat, deer, dog, horse, monkey, ship, and truck—as well as 100,000 unlabeled images drawn from a wide range of natural scenes. Although we do not use class labels in this work, the wide range of image diversity ensures a sufficiently varied embedding space, reducing the risk that neighborhood comparisons will represent an overly trivial manifold.

### 2.2. Embedding Model

To generate image embeddings, this study uses a ResNet-50 model trained on the ImageNet-1k [18] dataset. ResNet-50 is a deep convolutional neural network (CNN) constructed from residual convolution blocks organized with increasing channel depth and decreasing spatial resolution. It is known for its ease of optimization and its ability to mitigate accuracy degradation as network depth increases [16]. We selected this model for its ability to compress high-dimensional images into embeddings with low effective dimensionality [10]. Early layers of the ResNet-50 encode lower-level visual patterns, while deeper layers encode higher-level semantic structure. For each image, we collect a single embedding from the final convolutional block by removing the classification head and extracting the 2048-dimensional feature vector from the global average pooling layer. We computed embeddings in batches, where features are converted to NumPy arrays and concatenated into a single matrix for use in both the exact and approximate searches.

### 2.3. Sampling Strategy and Experimental Design

This research consists of multiple independent experiments designed to assess the divergence between exact and approximate neighborhoods. We perform numerous experimental runs, randomly sampling a fixed-size data subset without replacement from the full STL-10 dataset to reduce metric sensitivity and mitigate sampling bias. The dataset composition varies across runs; however, the embedding model, distance metric, ANN algorithm, and search effort parameters remain fixed across experimental conditions.

For each experimental run, we compute a new set of embeddings from the latest subset of data, reconstruct both exact and approximate indices, and use all embeddings in each subgroup as query points for both exact and approximate search. We evaluate each neighborhood’s properties over a predefined range of *k* values, using the same *k* for both exact and ANN searches. For each *k*, neighborhood metrics are computed across all queries and aggregated to produce a single per-run curve.

Furthermore, we average the resulting metric curves from each experiment and repeat this process multiple times to stabilize the aggregated curves. Each element of the experiment is performed in a controlled environment and is fully reproducible with implementation details provided in the accompanying code.

### 2.4. Exact Similarity Search

The purpose of using an exact similarity search is to establish an accurate gold-standard baseline for analyzing neighborhoods produced by ANN search. Although linear search becomes computationally infeasible as the dataset grows—even with just 100,000 image embeddings on most personal machines—it offers the highest achievable precision under unlimited computational resources. For this study, we performed an exact linear similarity search by computing true Euclidean distances from each embedding to all others, with zero approximation. This process produces a sorted list of exact distance-to-neighbor values for each query, serving as the structural baseline for all comparisons.

### 2.5. Approximate Similarity Search

The approximate nearest neighbor algorithm selected for this study is the Hierarchical Navigable Small World (HNSW) graph-based method. HNSW approximates the embedding space by organizing vectors into a multi-layered graph, enabling efficient navigation of high-dimensional spaces. Although we use Euclidean distance for both exact and approximate search, the underlying mechanisms differ substantially; the exact linear search evaluates all pairwise distances, whereas HNSW explores only a subset of items based on graph connectivity.

In HNSW, the extent of graph exploration during query-time search is governed by the search effort parameter, efSearch, which specifies the maximum size of the candidate list maintained during traversal. The neighborhood size *k* indicates the number of nearest neighbors retrieved for each query. Increasing efSearch raises the number of explored nodes, thereby enhancing neighborhood fidelity but requiring additional computational resources. The parameter efConstruction similarly controls the breadth of exploration during graph construction and affects the overall quality and connectivity of the ANN index. Unless otherwise specified, all references to *k*, efSearch, and efConstruction follow this notation throughout the manuscript.

To characterize how approximate neighborhood geometry depends on exploration behavior, we evaluate multiple controlled experimental regimes. The first regime examines fixed query-time search effort by holding M=32 and efConstruction=200 constant, while varying neighborhood size *k* over a set of fixed efSearch values {25,50,75,100}. This study demonstrates the point at which the ratio between *k* and efSearch leads to ANN failure. The second regime evaluates sensitivity to index construction by fixing M=32 and efSearch=50, while varying neighborhood size *k* across a set of fixed efConstruction values {50,100,200,300,400}. This configuration tests whether improved graph connectivity during construction prevents neighborhood drift and/or ANN failure at query time. In the final regime, we scale the query-time exploration factor proportionally with neighborhood size *k* by fixing M=32 and efConstruction=200 and setting efSearch=α×k, where α∈{0.25,0.50,1.0,2.0,4.0}. This study evaluates whether linear scaling of the exploration factor is sufficient to preserve neighborhood geometry and prevent ANN failure as *k* increases.

Across all configurations, we hold the ANN algorithm, distance metric, and evaluation measures constant, allowing observed differences in neighborhood structure to be explicitly attributed to search effort.

### 2.6. Evaluation Techniques

This study varies the neighborhood size *k* and the ANN configuration, and collects metrics on neighborhood overlap, average neighbor distance, normalized barycenter shift, and local intrinsic dimensionality (LID) to characterize how the approximate neighborhood structure depends on the search effort.

#### 2.6.1. Neighborhood Overlap

Neighborhood overlap measures the fraction of neighbors that the exact and ANN search agree on. It is equivalent to Recall@k, commonly used in approximate nearest neighbor evaluation, but is interpreted here as a measure of geometric agreement between neighborhoods rather than retrieval accuracy.

It returns a value between 0.0 and 1.0 representing the percentage of agreement, and is the most direct signal of neighborhood divergence: if an ANN perfectly reconstructs the true neighborhood, overlap is 1.0. We define this metric as

$$\mathrm{Neighborhood}\;\mathrm{Overlap}\left(i,k\right)=\frac{\left|\;N_{k}^{\mathrm{exact}}\left(i\right)\;\cap \;N_{k}^{\mathrm{ann}}\left(i\right)\;\right|}{k}$$

where $N_{k}^{\mathrm{exact}}\left(i\right)$ and $N_{k}^{\mathrm{ann}}\left(i\right)$ denote the sets of nearest neighbors *k* of the point *i* obtained using an exact and approximate search, respectively, and *k* is the size of the neighborhood.

#### 2.6.2. Average Neighbor Distance

Average neighbor distance is the average distance within a neighborhood of each query point to its kth nearest neighbor, for both exact and approximate searches. Even if neighborhood overlap is equal to 1.0, the average distance between neighbors can still degrade, and vice versa. Measuring average neighbor distance helps quantify degradation in the distribution of distances. For our purposes, we define average neighbor distance for both exact and approximate search queries as

$${\mathrm{AvgDist}}_{\mathrm{exact}}\left(k\right)=\frac{1}{N}\sum_{i=1}^{N}d_{i,k}^{\mathrm{exact}},\;{\mathrm{AvgDist}}_{\mathrm{ann}}\left(k\right)=\frac{1}{N}\sum_{i=1}^{N}d_{i,k}^{\mathrm{ann}}$$

where $d_{i,k}^{\mathrm{exact}}$ and $d_{i,k}^{\mathrm{ann}}$ represent the distance from point *i* to its kth nearest neighbor under exact and approximate search, respectively, and *N* is the total number of points in the dataset.

#### 2.6.3. Normalized Barycenter Shift

Barycenter shift quantifies how the center of a neighborhood shifts when comparing exact and ANN search. Each neighborhood has a barycenter, defined as the mean coordinate vector of its neighborhood embeddings. An ANN search may recover many of the same neighbors (high neighborhood overlap) and maintain similar distance scales (minimal distance distortion); however, it may still warp the estimated local geometry of the embedding space by incorporating neighbors from a different region. Barycenter shift captures this geometric drift: when the barycenters of the exact and approximate neighborhoods differ, it indicates that the HNSW-based ANN search is approximating the underlying manifold inconsistently.

Because the magnitude of barycenter shift depends on both the scale of the embedding space and the neighborhood radius, we normalize it to enable comparison across neighborhood sizes and experimental conditions. For a query point *i*, let $c_{k}^{\mathrm{exact}}\left(i\right)$ and $c_{k}^{\mathrm{ann}}\left(i\right)$ denote the barycenters of the exact and approximate *k*-nearest neighborhoods, defined as the mean of their respective neighbor embeddings. We define the normalized barycenter shift as

$$\mathrm{NormShift}\left(i,k\right)=\frac{{∥c_{k}^{\mathrm{exact}}\left(i\right)-c_{k}^{\mathrm{ann}}\left(i\right)∥}_{2}}{d_{i,k}^{\mathrm{exact}}}.$$

Here, $d_{i,k}^{\mathrm{exact}}$ denotes the distance from point *i* to its kth nearest neighbor under exact search. This normalization expresses barycenter displacement relative to the local neighborhood radius, yielding a scale-invariant measure of geometric drift.

#### 2.6.4. Local Intrinsic Dimensionality

Local intrinsic dimensionality (LID) measures how rapidly the neighborhood around a query point expands with increasing distance from the point, capturing the local geometry of the embedding space. Regions where distances expand rapidly have high LID, indicating many degrees of freedom; regions where distances grow slowly have low LID, indicating a collapsed or low-dimensional structure. Comparing the LID between exact and approximate searches within each neighborhood allows us to determine whether the ANN search distorts the local geometry of the manifold. We define this metric as

$$\hat{\mathrm{LID}}\left(i\right)=-{\left(\frac{1}{k-1}\sum_{j=1}^{k-1}\log\frac{d_{i,j}}{d_{i,k}}\right)}^{-1}$$

where $d_{i,j}$ is the distance from point *i* to its jth nearest neighbor, $d_{i,k}$ is the distance to the farthest neighbor in the *k*-NN set, and the ratio $d_{i,j}/d_{i,k}$ quantifies how rapidly the local neighborhood expands with distance.

LID estimates are known to be sensitive to neighbor ordering and small distance perturbations. For this reason, LID values are aggregated across all query points and averaged across experimental runs. Comparisons between exact and ANN search focus on consistent trends rather than absolute point-wise differences.

### 2.7. Implementation Details

All experiments for this study were conducted in a controlled environment using a dedicated workstation equipped with an NVIDIA RTX 4080 GPU (NVIDIA Corporation, Santa Clara, CA, USA). The GPU was used exclusively for embedding generation, as ResNet-50 contains more than 25 million parameters and benefits from accelerated convolution. All nearest neighbor computations were performed on the CPU using the FAISS library. We ran all experiments in a dedicated Conda environment using IPython version 8.37.0 with ipykernel version 7.1.0. All software dependencies are specified in a requirements.txt file to ensure reproducibility.

We computed image embeddings in batches and stored them in float32 format. Each experimental run produced an embedding matrix of shape 10,000×2048. For all experiments, we constructed the HNSW index with a connectivity parameter M=32. Index construction and query-time search parameters (efConstruction and efSearch) were varied according to the experimental condition, as described in Section 2.5.

### 2.8. Code Availability

All code used to conduct the experiments, generate data, and produce the reported results for this study, including embedding extraction, exact and approximate nearest neighbor search, and evaluation scripts, is publicly available at https://github.com/cooper-rm/CapacityLimitedFailureInANN (accessed on 9 January 2026).

## 3. Results

### 3.1. Fixed efSearch: Capacity-Limited Neighborhood Growth

This experiment examines how the quality of the approximate nearest neighbor (ANN) neighborhood degrades as the neighborhood size *k* increases, under fixed hyperparameters. We fix the Hierarchical Navigable Small World (HNSW) connectivity parameter M=32 and the construction effort efConstruction=200, and evaluate several fixed values of efSearch as the neighborhood size *k* grows. Under this regime, search effort (efSearch) remains constant as the number of neighbors queried increases. Fixing search effort exposes an inherent capacity mismatch: as *k* increases, the ANN search must identify increasingly distant neighbors without expanding the candidate set. We filter non-finite distance values before metric aggregation, which does not affect failure detection, defined by abrupt distance divergence rather than floating-point overflow.

**Figure 2:**
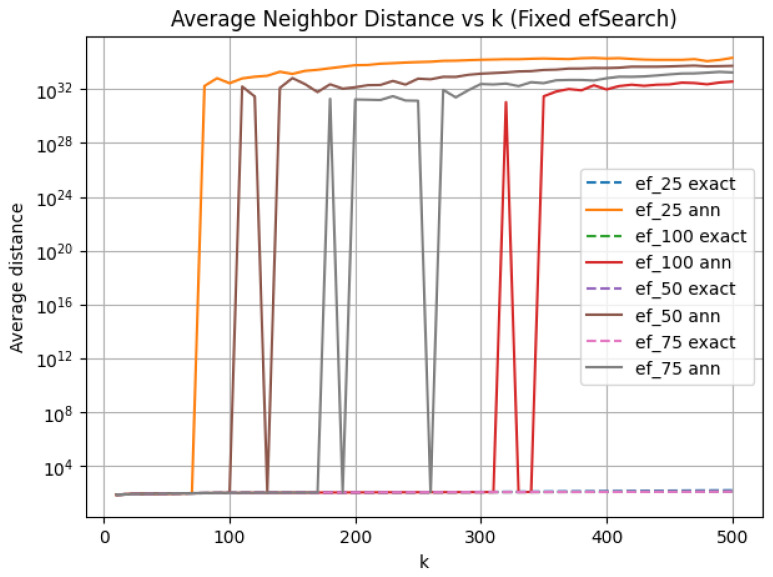
The average neighbor distance is plotted as a function of *k* for fixed efSearch, comparing exact and approximate nearest neighbor search. Dashed curves represent exact *k*-NN distances, while solid curves depict ANN distances obtained using HNSW. Different colors indicate distinct fixed values of efSearch. Catastrophic divergence is observed as an abrupt, orders-of-magnitude increase in the ANN distance curve relative to the corresponding exact curve, which signifies a hard algorithmic failure resulting from exhausted search capacity.

Across all fixed values, we observe a consistent failure pattern driven by distance error. When *k* is too large relative to efSearch, the ANN distance abruptly diverges from the exact distance, exhibiting extreme numerical inflation. This transition is discontinuous rather than gradual, indicating a hard algorithmic failure in which the search frontier is exhausted and no longer guided by meaningful metric comparisons. Although we observed neighborhood divergence before ANN failure, it was neither consistent nor reliable as an indicator of future algorithmic failure. However, we did find that ANN disruption (Figure 2) follows a capacity law: algorithmic failure consistently occurs when k≈2–3.5×efSearch. These results show that a fixed exploration factor imposes a hard upper bound on viable neighborhood size. Once *k* exceeds the upper bound of the efSearch capacity law, ANN search undergoes a qualitative failure characterized by invalid distances and unstable geometry.

Throughout this study, we define ANN failure as the abrupt, unbounded divergence in the average neighbor distance relative to exact search, characterized by extreme numerical inflation exceeding the maximum exact neighbor distance observed in the dataset. This criterion reflects a loss of numerical and geometric validity in the retrieved neighborhoods and is treated as a hard algorithmic failure, distinct from gradual degradation in neighborhood overlap or distance distortion.

**Figure 3:**
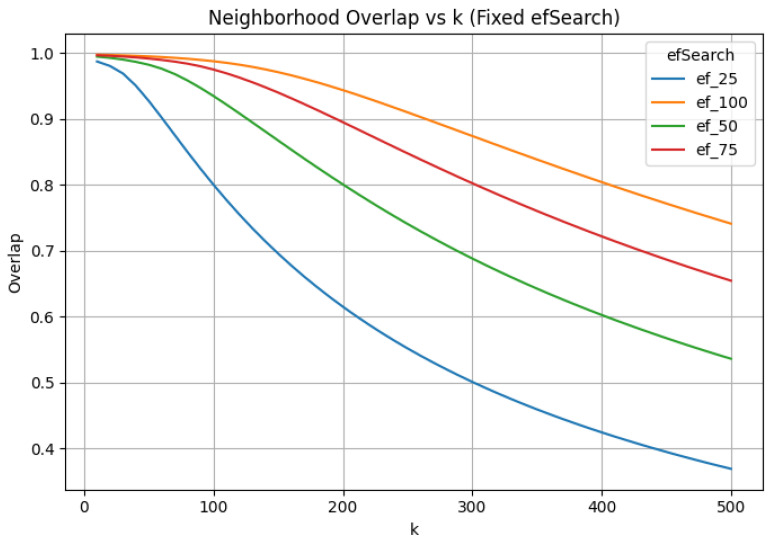
Neighborhood overlap between exact and approximate search as a function of *k* under fixed efSearch. Overlap degrades smoothly even beyond the point of distance failure, illustrating decoupling from metric validity.

After failure, ANN distances exceed even the maximum exact neighbor distance observed in the dataset. Once ANN fails, normalized barycenter shift, LID, and average neighbor distance are no longer meaningful, even if they remain computable. Overlap is reported (Figure 3) beyond this point only to illustrate its decoupling from true metric fidelity.

### 3.2. efConstruction Sensitivity: Index Quality vs. Search Capacity

**Figure 4:**
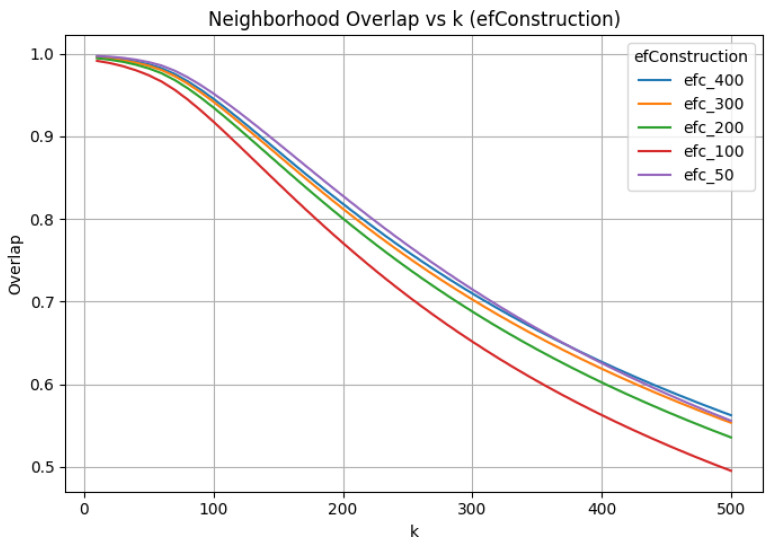
Neighborhood overlap between exact and approximate search as a function of *k* under varying efConstruction with fixed efSearch. Overlap degrades smoothly even beyond the point of distance failure, illustrating its decoupling from metric validity.

**Figure 5:**
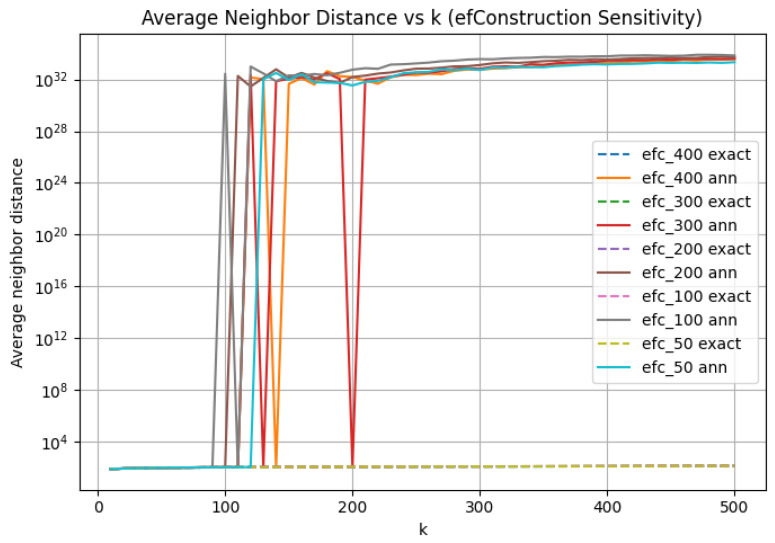
The average neighbor distance is plotted as a function of *k* for varying efConstruction and fixed efSearch, comparing exact and approximate nearest neighbor search. Dashed curves represent exact *k*-NN distances, while solid curves depict ANN distances obtained using HNSW. Different colors indicate distinct values of efConstruction. Catastrophic divergence is observed as an abrupt, orders-of-magnitude increase in the ANN distance curve relative to the corresponding exact curve, signifying a critical algorithmic failure resulting from exhausted query-time search capacity.

To show that a fixed exploration factor imposes a hard upper bound regardless of the quality of index construction, we performed a sensitivity study over a range of efConstruction values, holding M=32 and efSearch=50 fixed. Across all efConstruction settings, we observe qualitatively similar behavior as the neighborhood size increases. Higher efConstruction improves neighborhood quality, as reflected in generally higher neighborhood overlap as efConstruction increases (Figure 4). However, these improvements primarily delay rather than prevent a hard algorithmic failure (Figure 5).

Specifically, increasing efConstruction does not eliminate the existence of a failure boundary: distance disruption occurs at progressively larger *k* for higher efConstruction, but the transition remains abrupt and discontinuous. This behavior indicates that index quality can extend the usable range of *k* but cannot compensate for insufficient search effort. Once the search frontier is exhausted, even a well-constructed graph cannot prevent algorithmic failure.

### 3.3. α-Scaled Search Effort (efSearch=α×k)

Finally, we evaluate whether scaling search effort proportionally with neighborhood size can mitigate capacity-limited failures observed under fixed efSearch. For this experiment, we fix M=32 and efConstruction=200, and set the exploration factor as efSearch=α×k, subject to a small minimum value. We vary α across under-provisioned (α<1), matched (α=1), and over-provisioned (α>1) regimes.

Across all values of α, average neighbor distance remains the defining indicator of algorithmic validity. We find that ANN failure occurs immediately for small α (α=0.25), exhibiting extreme numerical inflation. Considering that hard failure follows a capacity law (failure occurs when k≈2−3.5×efSearch), and that the smallest neighborhood size evaluated is k=10, this regime necessarily violates the required search capacity, since efSearch<2−3.5×k.

At α=0.50, the ANN search remains algorithmically stable, and hard failure does not manifest across the evaluated range of *k*. As we systematically increase α to 1.0, we observe geometric stabilization across all evaluated metrics, including neighborhood overlap, average neighbor distance, barycenter shift, and local intrinsic dimensionality (LID). For α>1.0, we observe similar behavior between approximate and exact similarity search; increasing α beyond 1.0 continues to improve neighborhood fidelity (e.g., higher overlap and lower geometric drift), but with diminishing returns relative to the added search effort.

**Figure 6:**
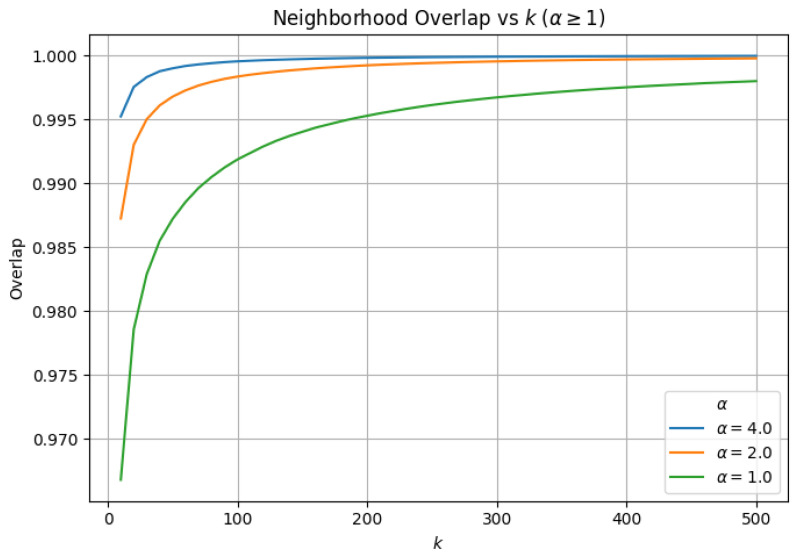
Neighborhood overlap between exact and approximate search as a function of *k* for α≥1 under α-scaled search effort (efSearch=α×k). Overlap remains consistently high and improves with increasing α, indicating stable neighbor identity recovery under proportional search scaling.

**Figure 7:**
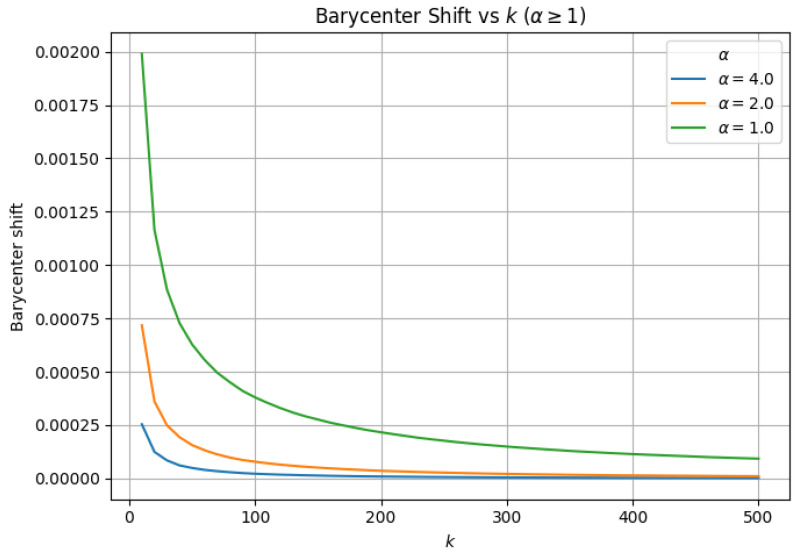
Barycenter shift between exact and approximate neighborhoods as a function of *k* for α≥1. Transient deviations at small *k* reflect finite-*k* effects, while rapid decay and long-range stability indicate preserved neighborhood geometry.

**Figure 8:**
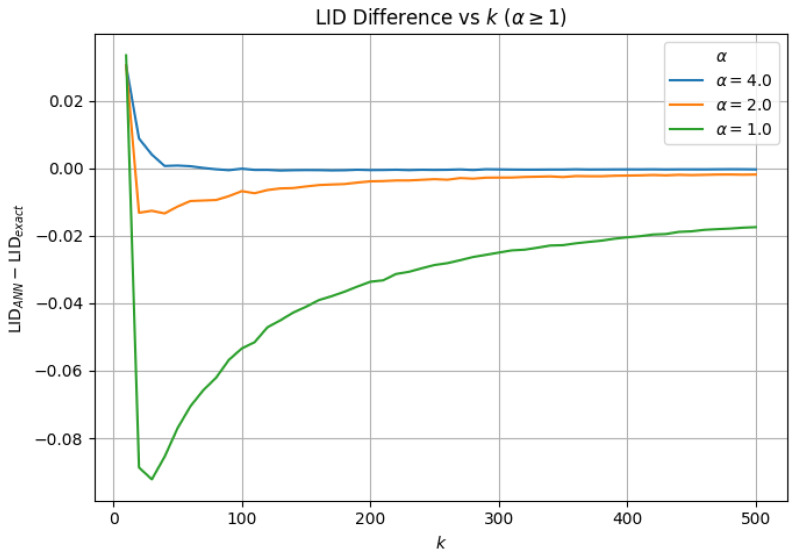
Local intrinsic dimensionality (LID) difference as a function of *k* for α≥1, reported as ${\mathrm{LID}}_{ANN}-{\mathrm{LID}}_{Exact}$. LID remains close to zero across *k* and improves with increasing α, suggesting that proportional search scaling preserves local manifold structure.

**Figure 9:**
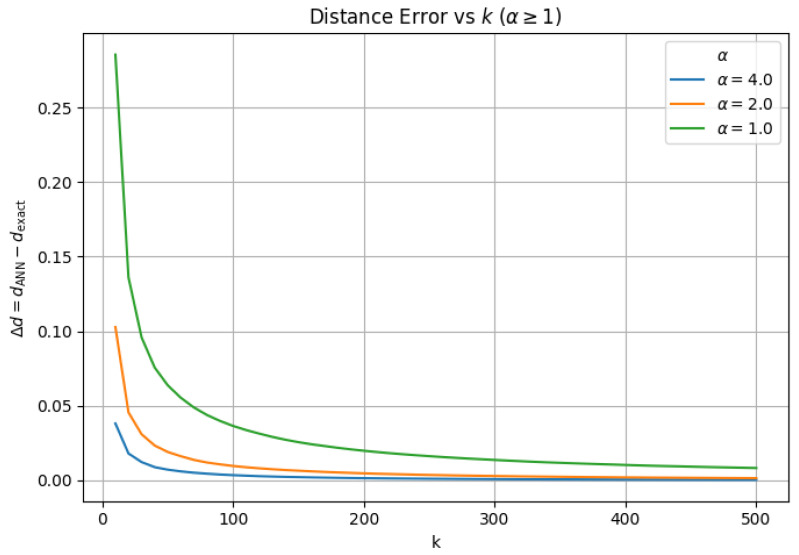
Average neighbor distance error $\Delta d=d_{\mathrm{ANN}}-d_{\mathrm{exact}}$ as a function of *k* for α≥1. Error remains small and decays with *k*, confirming stable ANN behavior when search effort scales proportionally with neighborhood size.

To demonstrate geometric stability in this regime, we report neighborhood overlap (Figure 6), barycenter shift (Figure 7), LID (Figure 8), and average neighbor distance error (Figure 9) only for α>1.0, where ANN distance remains well-defined. For α>1.0, all metrics exhibit transient deviations at small neighborhood sizes due to finite-sample effects, but rapidly stabilize as *k* increases, with no evidence of hard failure or geometric collapse.

Local intrinsic dimensionality (LID) is particularly sensitive to capacity-limited search behavior because it relies on the relative ordering and spacing of neighbors within a local region, rather than solely on neighbor identity. When approximate nearest neighbor (ANN) search operates within its capacity limits, the relative growth of distances within each neighborhood is maintained, resulting in LID estimates that closely align with those from exact search. However, if search capacity is inadequate, approximate neighborhoods may contain spurious distant points or exclude intermediate neighbors, which distorts the local distance distribution and biases LID estimates, even when neighborhood overlap remains high. Therefore, LID serves as a complement to overlap- and distance-based metrics by assessing whether the local expansion geometry of the embedding manifold is preserved, rather than simply determining if similar points are retrieved.

## 4. Discussion

The primary failure mode of approximate nearest neighbor (ANN) search is identified as a capacity-limited, discontinuous breakdown, rather than a gradual geometric drift. The principal driver of algorithmic failure is insufficient search effort relative to neighborhood size. Across all experiments, average neighbor distance consistently served as the main indicator of algorithmic validity. When the ANN distance diverges sharply from the exact distance, the search process ceases to produce meaningful neighborhoods. This abrupt failure is governed by a straightforward capacity law that relates neighborhood size *k* to search effort efSearch.

These findings have significant implications for systems that rely on embedding-space neighborhoods, including image retrieval, semantic search, clustering, and manifold analysis. ANN search demonstrates high reliability within its capacity envelope, but may experience catastrophic failure if this threshold is surpassed. Importantly, such failure is abrupt and may not be detectable using only overlap-based metrics. Documenting this behavior is relevant for scientific applications that approximate embedding spaces and offers practical guidance regarding when ANN search can be used interchangeably with exact *k*-NN and when geometric differences become substantial.

Several limitations are associated with the data and embedding model choices in this study. Only a single embedding model (ResNet-50) and one dataset (STL-10) were utilized, each with specific structural properties that may influence the observed behavior. The embedding dimensionality was fixed at 2048 and not varied, so the dependence of failure behavior on embedding dimension or representation type was not examined.

A further limitation pertains to the algorithmic and parameter scope of the analysis. Only one ANN algorithm, Hierarchical Navigable Small World (HNSW), was evaluated. Although HNSW is widely used, alternative indexing schemes such as IVF-PQ, ScaNN, and locality-sensitive hashing (LSH) may demonstrate different capacity limits or failure modes. Furthermore, while multiple hyperparameter regimes were explored, the analysis does not comprehensively characterize all possible index configurations.

The analysis is limited to geometric fidelity and numerical stability, without direct consideration of semantic consistency. Although geometric neighborhood collapse clearly indicates algorithmic failure, it remains unresolved whether such failures correspond to semantic degradation in downstream tasks. The relationship between geometric approximation error and semantic retrieval quality requires further investigation.

Future research should extend this analysis to larger-scale datasets, alternative embedding models, and additional ANN algorithms to assess the generalizability of the observed capacity law. Investigating how failure thresholds scale with intrinsic dimensionality and dataset size would further clarify the relationship among approximation, geometry, and search capacity. More broadly, these findings highlight the importance of evaluating ANN systems not only by Recall@k but also by numerical and geometric stability across diverse operating regimes.

## Data Availability

The data used in this study are derived from the publicly available STL-10 dataset. All code used to generate embeddings, perform exact and approximate nearest neighbor search, and compute evaluation metrics is publicly available at https://github.com/cooper-rm/CapacityLimitedFailureInANN, accessed on 5 December 2025.
